# *GmbZIP152*, a Soybean bZIP Transcription Factor, Confers Multiple Biotic and Abiotic Stress Responses in Plant

**DOI:** 10.3390/ijms231810935

**Published:** 2022-09-19

**Authors:** Mengnan Chai, Rongbin Fan, Youmei Huang, Xiaohu Jiang, Myat Hnin Wai, Qi Yang, Han Su, Kaichuang Liu, Suzhuo Ma, Zhitao Chen, Fengjiao Wang, Yuan Qin, Hanyang Cai

**Affiliations:** 1 Fujian Provincial Key Laboratory of Haixia Applied Plant Systems Biology, State Key Laboratory of Ecological Pest Control for Fujian and Taiwan Crops, College of Agriculture, Fujian Agriculture and Forestry University, Fuzhou 350002, China; 2 College of Life Science, Fujian Agriculture and Forestry University, Fuzhou 350002, China; 3 State Key Laboratory for Conservation and Utilization of Subtropical Agro-Bioresources, Guangxi Key Lab of Sugarcane Biology, College of Agriculture, Guangxi University, Nanning 530004, China; 4 Pingtan Science and Technology Research Institute, Fujian Agriculture and Forestry University, Fuzhou 350400, China

**Keywords:** soybean, *GmbZIP152*, *S. sclerotiorum*, salt, drought, heavy meta

## Abstract

Soybean is one of the most important food crops in the world. However, with the environmental change in recent years, many environmental factors like drought, salinity, heavy metal, and disease seriously affected the growth and development of soybean, causing substantial economic losses. In this study, we screened a bZIP transcription factor gene, *GmbZIP152*, which is significantly induced by *Sclerotinia sclerotiorum* (*S. sclerotiorum*), phytohormones, salt-, drought-, and heavy metal stresses in soybean. We found that overexpression of *GmbZIP152* in *Arabidopsis* (*OE-GmbZIP152*) enhances the resistance to *S. sclerotiorum* and the tolerance of salt, drought, and heavy metal stresses compared to wild-type (WT). The antioxidant enzyme related genes (including *AtCAT1*, *AtSOD,* and *AtPOD1*) and their enzyme activities are induced by *S. sclerotiorum*, salt, drought, and heavy metal stress in *OE-GmbZIP152* compared to WT. Furthermore, we also found that the expression level of biotic- and abiotic-related marker genes (*AtLOX6*, *AtACS6*, *AtERF1*, and *AtABI2,* etc.) were increased in *OE-GmbZIP152* compared to WT under *S. sclerotiorum* and abiotic stresses. Moreover, we performed a Chromatin immunoprecipitation (ChIP) assay and found that GmbZIP152 could directly bind to promoters of ABA-, JA-, ETH-, and SA-induced biotic- and abiotic-related genes in soybean. Altogether, *GmbZIP152* plays an essential role in soybean response to biotic and abiotic stresses.

## 1. Introduction

Soybean is famous for its high content of oil and protein over the world. It is one of the most critical dicot crops and the primary source of edible vegetable oil and high-protein livestock feed [1]. However, many biotic and abiotic stresses seriously affect its growth and development [2,3]. Plants have evolved complex signaling transduction pathways and mechanisms to survive in extreme environments [4,5,6]. Transcription factors (TFs) affect the tolerance to abiotic and biotic stresses through interacting with *cis*-elements in the promoter region of downstream genes to activate or repress their expression [7]. The basic leucine zipper (bZIP) transcription factor is a large TF family in the plant. Members of bZIP have two conserved structures, (1) A DNA-binding region that includes 18 amino acid residues and contains an invariant motif N- × 7- R/K- × 9. (2) A leucine zipper region, used for recognition and dimerization, is composed by a heptad repeat of leucine or other bulky hydrophobic amino acids, which creates an amphipathic helix [8,9].

*bZIP* genes play essential roles in many biological processes, including plant growth, development, and flowering [10,11,12] in soybean. The bZIP transcription factor FDc1 affects node number, plant height, and flowering time by physically interacting with Dt1 in soybean [13]. GmbZIP5, as the additional cofactor of GmMYB176, controls isoflavonoid biosynthesis in soybean [14]. GmFT2a and GmFT5a induce the expression of floral identity genes in soybean through physical interaction with and transcriptional upregulation of the bZIP TF GmFDL19 [15]. bZIP proteins are also involved in biotic and abiotic stress responses. *GmbZIP2*, a drought stress-related gene in soybean, overexpression can enhance drought tolerance and salt tolerance in transgenic *Arabidopsis* via improving the expression of the stress-responsive genes *GmMYB48*, *GmWD40*, *GmDHN15*, *GmGST1* and *GmLEA* [16]. *GmbZIP15* plays a positive role in pathogen resistance in soybean by relying on phytohormone signaling [17]. In addition, the overexpression of *GmbZIP15* in soybean could reduce the tolerance to abiotic stresses associated with declined expression of stress-related genes, defective stomatal aperture regulation, and lower antioxidant enzyme activities [18]. GmbZIP19 can regulate disease defense and abiotic stress tolerance as a multi-functional TF in *Arabidopsis* [19]. We analyzed 160 full-length bZIP genes from soybean and found that at least 75.6% of bZIP genes displayed transcriptional changes after drought and flooding treatment [20]. Among these genes, the expression of *GmbZIP152* was induced by multiple stress responses and has not been investigated yet [20].

In this study, we identified and cloned the *GmbZIP152* from soybean. The expression profile indicated that the expression of *GmbZIP152* was induced by *S. sclerotiorum* infection and the treatment of salt, drought, and heavy metal stresses. Furthermore, we found that *OE-GmbZIP152* enhanced the resistance of *S. sclerotiorum* and the tolerance of salt, drought, and heavy metal stresses compared to wild-type (WT). In summary, our results verified that *GmbZIP152* plays an important role in biotic and multiple abiotic stress responses. These results reveal that the *GmbZIP152* gene may be necessary for developing and increasing production in soybean plants under long-term stress conditions.

## 2. Results

### 2.1. Bioinformatics Analysis of GmbZIP152

*GmbZIP152* cDNA consists of 1266 bp (Figure S1A) and encodes a protein with a conserved bZIP domain (Figure S1B). The relative molecular mass is 16.93 kDa, and the theoretical isoelectric point (pI) is 5.21. According to the gene structure and conserved motif analyses, the genes without intron are classified into the subgroup S [9]. *GmbZIP152* was categorized into subgroup S and has not been functionally characterized. The homologs of GmbZIP152 are GmbZIP33 from soybean (*Glycine max*), OsbZIP38 from rice (*Oryza sativa*), and AtbZIP53 from *Arabidopsis* (*Arabidopsis thaliana*) (Figure S1C). Homology analysis shows that they share a conserved bZIP DNA-binding domain and a leucine zipper dimerization motif. The basic DNA binding region is conserved and contains a 52-amino acid long basic region (N-x7-R/K-x9). They all belong to the members of subgroup S. Among them, GmbZIP33 might be involved in the processes of abiotic stress [21]. OsbZIP38 was a molecular switch in low-temperature signaling [22]. AtbZIP53 was involved in the regulation of plant responses to abiotic stresses by affecting the transcriptional activation of proline dehydrogenase (ProDH), which was catalyzing the first step in proline degradation [23]. These researches suggest that *GmbZIP152* possesses a potential function in response to abiotic stress.

### 2.2. Expression Profile of GmbZIP152 in Response to Various Stresses

Considering the potential involvement of *GmbZIP152* in stress responses, we investigated the distribution of stress-related *cis*-elements in their promoter regions (2.5 kb region upstream of the transcription start site) using PlantCARE (http://bioinformatics.psb.ugent.be/webtools/plantcare/html/, accessed on 30 January 2020). *GmbZIP152* possessed five stress response elements, G-box recognition site (CACGAC), involved in light-responsive element [24]; TC-rich repeat (G/ATTCTCT), involved in defense and stress response [25]; MYB (CAACTG), involved in drought-inducibility [26]; TCA-element (CCATCTTTTT), involved in salicylic acid responsiveness [27]; and CGTCA-motif (CGTCA), involved in the MeJA-responsiveness [28] (Figure S1D), indicating that expression of *GmbZIP152* is associated with the biotic and abiotic stresses in plant development.

**Figure 1:**
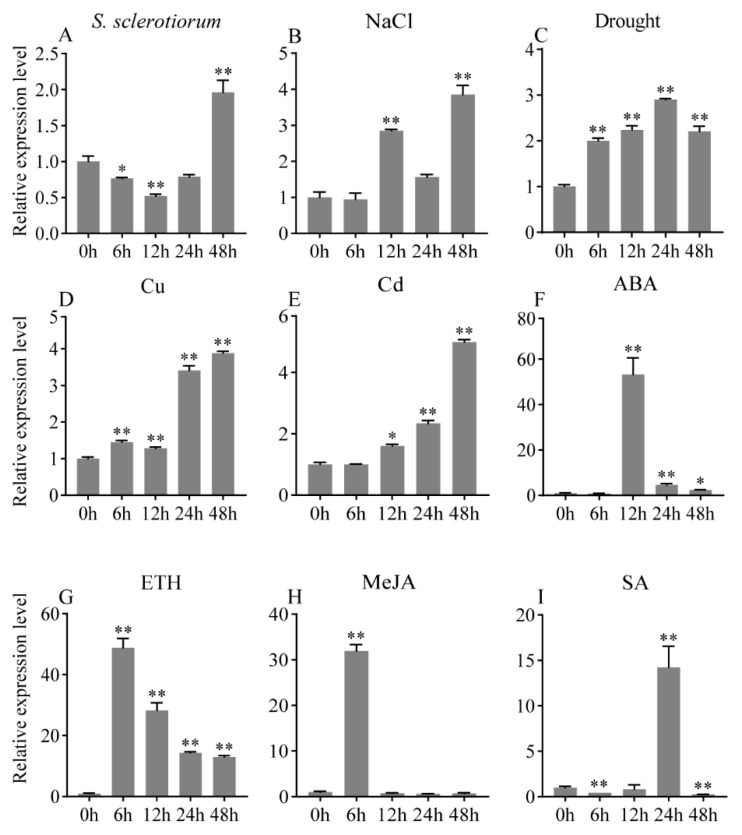
Expression pattern of the *GmbZIP152* gene revealed by quantitative PCR analysis from different treatments in soybean. (**A**) Response of *GmbZIP152* to pathogens infection in WT and *GmbZIP152* transcript levels were detected by qPCR with *Sclerotinia. sclerotiorum* (*S. sclerotiorum*) infection at the different time points in soybean (**B**–**E**) *GmbZIP152* expression in response to various abiotic stress treatments (150 mM NaCl, 400 mM Mannitol, 150 μM CuSO_4_, and 150 μM CdSO_4_). (**F**–**I**) *GmbZIP152* expression in response to various hormone treatments [1.0 μM Abscisic acid (ABA), 150μM Methyl jasmonic acid (MeJA), 400 μM Ethylene (ETH), and 250 μM Salicylic acid (SA)]. Errors bars indicate ± SD of three biological replicates. Asterisks indicate significant differences for the indicated comparisons based on a Students’ *t*-test (** *p* < 0.01; 0.01 < * *p* < 0.05).

Two-week-old soybean seedlings were treated with various stresses, and the leaves were used to further explore and evaluate the function of *GmbZIP152* using qRT-PCR. Our results showed that the expression of *GmbZIP152* increased strikingly in response to all tested stimuli (Figure 1). Specifically, the expression of *GmbZIP152* increased dramatically after hormone stimulation (Figure 1F–I). At the same time, the peak appeared at 12 h after ABA treatment (Figure 1G,H), 6h after ETH and MeJA treatment, and 24h after SA treatment. After the infection of *S. sclerotiorum*, the transcript level of *GmbZIP152* repressed significantly within the first 24 but increased at 48 h (Figure 1A). Meanwhile, under salt treatment, the expression of *GmbZIP152* increased within 12 h, followed by a decrease, and reached its maximum at 48 h. Further, *GmbZIP152* mRNA accumulated and reached a maximum level of 24 h under drought stress. Copper and cadmium stresses induced *GmbZIP152* transcripts and reached a maximum level at 48 h (Figure 1D,E). In addition, the expression of *GmbZIP152* also was induced by NaCl, mannitol, heavy metals and hormones in the mature soybean leaves (Figure S2). These findings indicated that *GmbZIP152* might regulate multiple stresses during soybean development.

### 2.3. OE-GmbZIP152 Enhances Resistance to S. sclerotiorum Infection in Arabidopsis

**Figure 2:**
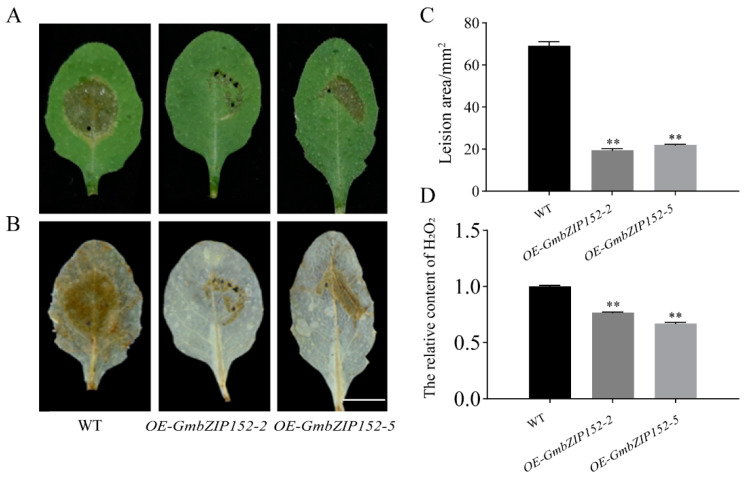
Biotic stress analysis of *GmbZIP152* transgenic *Arabidopsis* plants in response to *Sclerotinia. sclerotiorum* (*S. sclerotiorum*). (**A**,**B**) Phenotype observation of *GmbZIP152* transgenic plants in response to *S. sclerotiorum* and Diaminobenzidine (DAB) staining. Bar = 1 cm. (**C**) Lesion area measurement. (**D**) The relative content H_2_O_2_. *GmbZIP152* transgenic *Arabidopsis* plants (*OE-GmbZIP152-2* and *OE-GmbZIP152-5*, two independent transgenic lines). The error bars indicate ± SD (n = 3 replicates). Asterisks indicate significant differences for the indicated comparisons based on a Students’ *t*-test (** *p* < 0.01).

In this experiment, we did the pathogenicity assay to investigate the *GmbZIP152* gene response to the pathogen. The rosette leaves of three-week-old wild-type (WT) and overexpressed *GmbZIP152* transgenic *Arabidopsis* plants (*OE-GmbZIP152-2* and *OE-GmbZIP152-5*, two independent transgenic lines, *OE-GmbZIP152*) were inoculated with the same concentration of *S. sclerotiorum* for 12 h. After the inoculation treatment, we observed and calculated the relative lesion areas of infected leaves with Image J software. The results showed that the leaves of *OE-GmbZIP152* plants significantly increased resistance to *S. sclerotiorum* than the WT (Figure 2A,C).

Plants will produce a large amount of ROS under biotic and abiotic stresses, and the increase of ROS like H_2_O_2_ and O^2−^ can significantly damage plant cells [17,29]. To confirm the biotic stress tolerance in *OE-GmbZIP152* under *S. sclerotiorum* treatment, we used DAB staining to visualize H_2_O_2_ accumulation in three-week-old *OE-GmbZIP152* and WT leaves after pathogen infection. And the *OE-GmbZIP152* and WT leaves were decolorized using 75% alcohol (Figure 2B). In leaves of *OE-GmbZIP152* plants, brown precipitates were substantially less than WT after being infected with fungus. The H_2_O_2_ content can indicate the degree of leaf damage (Figure 2D) [17]. The result indicates that *OE-GmbZIP152* plants improved the tolerance to *S. sclerotiorum*.

### 2.4. OE-GmbZIP152 Confers Salt, Drought, Heavy Metal Tolerance and Decreased Sensitivity to Plant Hormones in Arabidopsis

**Figure 3:**
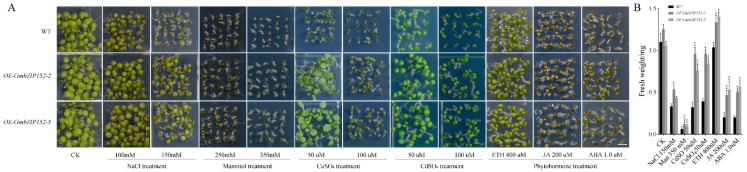
Phenotypic analysis of *GmbZIP152* transgenic *Arabidopsis* plants in response to salt, drought, heavy metal, plant hormones treatment in *Arabidopsis*. (**A**) All the seeds were germinated on the 1/2 Murashige and Skoog Medium (MS) medium under normal conditions or supplemented with CK (Control check), NaCl (100 mM and 150 mM), mannitol (250 mM and 350 mM), CuSO_4_ (50 uM and 100 uM), CdSO_4_ (50 uM and 100 uM), ETH (400 uM), JA (200 uM), and ABA (1.0 uM) for 1 week (Scale bar, 1 cm). (**B**) Calculation of the seedlings’ fresh weights. *GmbZIP152* transgenic *Arabidopsis* plants (*OE-GmbZIP152-2* and *OE-GmbZIP152-5*, two independent transgenic lines). Abscisic acid (ABA), Methyl jasmonic acid (MeJA), Ethylene (ETH), and Salicylic acid (SA). Asterisks indicate significant differences for the indicated comparisons based on a student’s *t*-test (** *p* < 0.01; 0.01 < * *p* < 0.05).

The expression pattern of *GmbZIP152* suggested that *GmbZIP152* may play an important role in multiple stresses. To examine whether *GmbZIP152* is involved in the processes of plant stress response, we handled *OE-GmbZIP152* plants with three stress treatments (salt, drought, and heavy metals). The seeding of *OE-GmbZIP152* and WT were planted in 1/2 MS media as the control group. We used the concentration of 100 and 150 mM NaCl to simulate salt treatment. Then, the medium containing 250 and 350 mM mannitol mimics drought treatment. CuSO_4_ and CdSO_4_ simulated heavy metal treatment. The results showed that there were no noticeable differences in the phenotype between *OE-GmbZIP152* plants and WT plants in the control condition. However, when the *OE-GmbZIP152* plants and WT plants were exposed to NaCl, mannitol, and heavy metal, the WT seedlings were severely repressed by all treatments compared to *OE-GmbZIP152* plants (Figure 3). At the same time, the fresh weight (Figure 3B) and root length (Figure S3B) of WT were significantly decreased under 150 mM NaCl, 350 mM mannitol, 50 uM CuSO_4_, and 50 uM CdSO_4_ treatment. In addition, we continuously watered the three-week-old plants with 150 mM NaCl, 350 mM mannitol, 100 uM CuSO_4_, and 100 uM CdSO_4_ for 18 days. It was found that the leaves of WT plants gradually lost greenness, and the growing situation was severely inhibited (Figure S4). These results suggest that *OE-GmbZIP152* seedlings showed higher tolerance than WT plants when it was exposed to salt, drought, and heavy metal stress.

To further assess the response of *GmbZIP152* to plant hormone, seeds of *OE-GmbZIP152-2* and *OE-GmbZIP152-5* were planted on 400 uM ETH, 200 uM JA, and 1.0 uM ABA 1/2 MS agar medium for seven days. Under normal conditions, *OE-GmbZIP152* plants were not different from WT. However, after being exposed to exogenous ETH, JA, and ABA, the transgenic plants suffered less impairment than WT plants. The root length and fresh weight of WT seedlings were decreased compared with *OE-GmbZIP152* (Figure 3B and Figure S3B). These results showed that *OE-GmbZIP152* plants are less sensitive than WT to plant hormones.

### 2.5. OE-GmbZIP152 Enhances Antioxidant Enzyme in Arabidopsis

**Figure 4:**
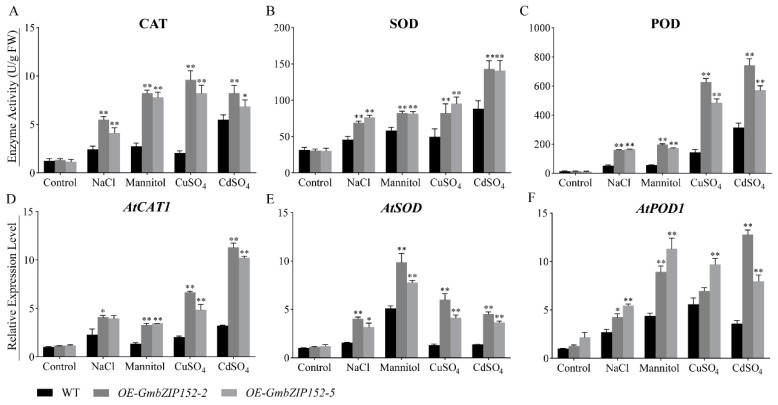
ROS scavenges enzyme activities of *GmbZIP152* transgenic *Arabidopsis* plants under salt, drought, and heavy metal stresses. (**A**) catalase (CAT), (**B**) superoxide dismutase (SOD), and (**C**) peroxidase (POD) enzyme activity was directly determined from fresh leaves. The relative expression level of (**D**) *AtCAT1*, (**E**) *AtSOD*, and (**F**) *AtPOD* was analyzed by qRT-PCR. The error bars indicate ± SD (n = 3 replicates). *GmbZIP152* transgenic *Arabidopsis* plants (*OE-GmbZIP152-2* and *OE-GmbZIP152-5*, two independent transgenic lines). Asterisks indicate significant differences for the indicated comparisons based on a student’s *t*-test (** *p* < 0.01; 0.01 < * *p* < 0.05).

To confirm the abiotic stress tolerance in transgenic lines under salt, drought, and heavy metal treatment for 24 h, we determined the activities of the three main antioxidant enzymes involved in ROS scavenging, including catalase (CAT), superoxide dismutase (SOD), and peroxidase (POD). Under these treatments, the enzyme activities of CAT, SOD, and POD in *OE-GmbZIP152* plants were significantly higher than those of WT plants (Figure 4A–C). In addition, the mRNA levels of *AtCAT1*, *AtSOD*, and *AtPOD1* of *OE-GmbZIP152* plants were also significantly higher than WT (Figure 4D–F). These results demonstrate that overexpression of *GmbZIP152* improves resistance to salt, drought, and heavy metal stresses by increasing the expression levels of antioxidant enzyme corresponding genes.

### 2.6. The Transcription Levels Analysis of Stress-Related Genes in OE-GmbZIP152 and WT Plants under Biotic and Abiotic Stresses

**Figure 5:**
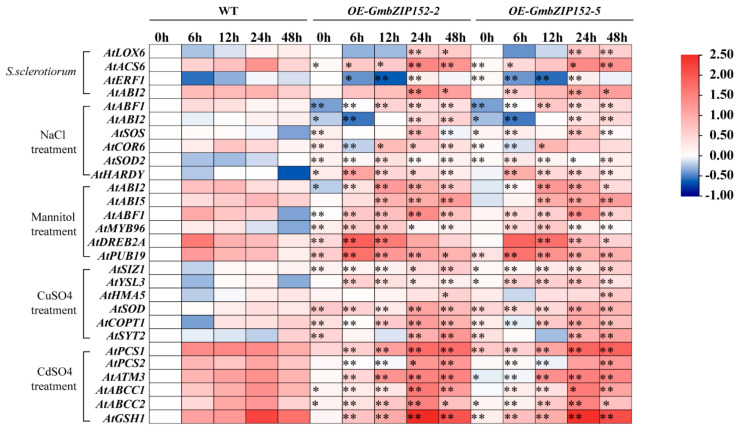
The expression profiles of stress−related genes in *GmbZIP152* transgenic *Arabidopsis* plants and WT under disease, salt, drought, and heavy metal stresses. Heat−map was constructed from relative gene expression levels (qRT−PCR) under different stresses using TBtools. *Sclerotinia. sclerotiorum* (*S. sclerotiorum*), *GmbZIP152* transgenic *Arabidopsis* plants (*OE−GmbZIP152−2* and *OE−GmbZIP152−5*, two independent transgenic lines). The stars indicate that the expression of genes is different in *OE−GmbZIP152* compared with WT at the same time. Asterisks indicate significant differences for the indicated comparisons based on a student’s *t*-test (** *p* < 0.01; 0.01 < * *p* < 0.05).

To further understand the causal factor behind the *S. sclerotiorum*, salt-, drought-, and heavy metal stresses high tolerance of *OE-GmbZIP152* plants, we investigated the relative expression level of several known disease- (*AtLOX6*, *AtACS6*, *AtERF1*, and *AtABA2*) [30,31,32], salinity- (*AtABF1*, *AtABI2*, *AtSOS*, *AtCOR6*, *AtSOD2*, and *AtHARDY*) [33,34,35], drought- (*AtABI2*, *AtABI5*, *AtABF1*, *AtMYB96*, *AtDREB2A*, *AtPUB19*) [36,37], copper- (*AtSIZ1*, *AtYSL3*, *AtHMA5*, *AtSOD*, *AtCOPT1*, and *AtSYT2*) [38,39], and cadmium- (*AtPCS1*, *AtPCS2*, *AtATM3*, *AtABCC1*, *AtABCC2*, and *AtGSH1*) [40] responsive genes in three-week-old leaves of transgenic lines and WT by qRT-PCR, heatmap were generated based on qRT-PCR results. Our data proved that stresses-responsive gene expression levels were upregulated in leaves of transgenic lines when exposed to these stresses and higher than in WT (Figure 5). According to the analytical data from the above experiments, *GmbZIP152* overexpressed could increase the tolerance of disease, salt, drought, and heavy metal stresses by upregulating biotic and abiotic stress relative genes.

### 2.7. Transient Expression of GmbZIP152 Induces High Expression of Stress-Related Genes

**Figure 6:**
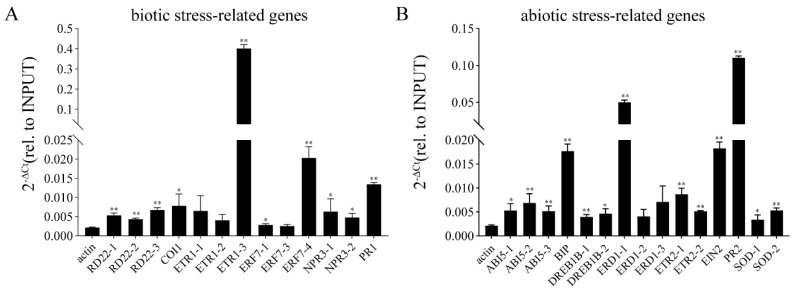
The Chromatin immunoprecipitation (ChIP) result of *GmbZIP152* transient expressing soybean. (**A**) ChIP-qPCR analysis of GmbZIP152 binding to biotic stress-related genes using GFP antibody and *35S-GmbZIP152-GFP* transient expressing soybean. (**B**) ChIP-qPCR analysis of GmbZIP152 binding to abiotic stress-related genes using GFP antibody and *35S-GmbZIP152-GFP* transient expressing soybean. Three independent biological replicates were performed. The error bars indicate ± SD (n = 3 replicates). Asterisks indicate significant differences for the indicated comparisons based on a student’s *t*-test (** *p* < 0.01; 0.01 < * *p* < 0.05).

In the *GmbZIP152* soybean resistance pathway, Chromatin immunoprecipitation (ChIP) was performed using *35-GmbZIP152-GFP* transient expression in two-week-old soybean. The transient overexpression levels of *GmbZIP152* were examined using qRT-PCR (Figure S5). We examined the changes in the expression of biotic and abiotic stress-related genes. The biotic stress-related genes include the *GmNPR3* and *GmPR1*, *GmCOI1*, *GmETR1*, *GmERF7*, and *GmRD22* (Figure 6A). The abiotic stress-related genes include *GmABI5*, *GmBIP*, *GmDREB1B*, *GmERD1*, *GmETR2*, *GmEIN2*, *GmPR2*, and *GmSOD* (Figure 6B). They were also related to ABA (*GmRD22* and *GmABI5*), JA (*GmCOI1*), ETH (*GmETR1*, *GmETR2*, *GmERF7*, and *GmEIN2*), and SA (*GmNPR3*, *GmPR1*, and *GmPR2*) signaling pathways. We designed the primers at both ends of the *cis*-acting element G-box of the relevant gene promoter, and ChIP-qPCR detected the expression levels of related genes. The results showed that the relative transcript levels of *GmERD1*, *GmEIN2*, *GmPR2*, and *GmETR1*, increased continuously during transient expression of *GmbZIP152*. This indicated that transient *GmbZIP152* overexpression could enhance the resistance to disease infection and tolerance of abiotic stresses.

## 3. Discussion

Soybean is broadly used as edible oil, animal feed protein concentrates, and various industrial products. Climate variability has a big impact on crop yields [41]. Soybean production significantly losses yearly due to biotic and abiotic stresses during the growth process [42]. Nowadays, the human population is large and large, and the environmental condition has changed daily; therefore, improving soybean yield quality is an urgent problem to solve. The bZIP gene family is one of the largest transcription factor families in the plant. Many studies have shown the bZIP family of different crops like rice [43], soybean [16], cotton [44], and maize [45] have different ways of responding to biotic and abiotic stresses. In this study, we identified and cloned the *GmbZIP152* gene from soybean and analyzed the function of this gene.

Transcriptional factors regulate the transcription of downstream genes through binding to *cis*-element in the promoter region. For example, *bHLH106* confers salt tolerance on *Arabidopsis* by directly binding to the G-box in the target genes [24]. MYB recognition site is the binding site for the MYB transcription factor, which is involved in plant disease stress [46]. Our results showed that the promoter of *GmbZIP152* has an abundance of stress-responsive cis-element in the 2500 bp promoter region, such as G-Box recognition site, MYB recognition site, TC-rich repeats, TCA-element, and CGTCA-motif (Figure S1). *Cis*-elements analysis suggests that *GmbZIP152* may be regulated by disease defense, salt, drought, and other stress responses. The expression changes of *GmbZIP152* in soybean leaves under different biotic and abiotic treatments were evaluated to test this hypothesis. Our result showed that *GmbZIP152* responses to salt, drought, heavy metal (CuSO_4_ and CdSO_4_) stresses, and phytohormones (ABA, ETH, MeJA, and SA) (Figure 1), suggesting that *GmbZIP152* may involve in stress responses. Therefore, to clarify the potential function of *GmbZIP152* in response to different stresses, we overexpressed *GmbZIP152* in *Arabidopsis* and revealed that *OE-GmbZIP152* plants increased resistance to *S. sclerotiorum*, high salinity, drought, and heavy metal, significantly.

Plant growth is greatly affected by combinate environmental stresses such as diseases, high salt, drought, and heavy metal. To adapt to the environment, plants derive several strategies, including the induction of antioxidant enzymes, plant hormones, and regulatory genes [47]. Previous research has shown that ETH and JA signaling pathways are considered the main pathways for plants to resist biological invasion, and ABA participates in the immune response of plants via regulating the ET/JA signaling pathway [48]. Overexpression of jasmonate-responsive *OsbHLH034* can increase the tolerance to bacterial blight in *rice* [49]. The expression of *GmbZIP152* in WT soybean was induced by ABA, SA, JA, and ETH (Figure 1F–I), and *OE-GmbZIP152* plants were less sensitive to exogenous hormones than WT (Figure 3). We detected the expression of marker genes for multiple hormones in *OE-GmbZIP152* and WT plants under *S. sclerotiorum* treatment. Among these genes, *AtLOX6*, a JA biosynthetic gene, has been reported to function in response to stress resistance [32]. ERF1 and ABI2, which participate in the ETH or ABA signaling pathways, were upregulated in *OE-GmbZIP152* leaves upon *S. sclerotiorum* inoculation. These further confirmed the involvement of phytohormone signaling in regulating *GmbZIP152*-mediated pathogen resistance. Numerous studies have shown that hormones are essential in the process of bZIP transcription factors improving tolerance to abiotic stress. Overexpression of *StbZIP65* in potato (*Solanum tuberosum* L.) enhanced salt tolerance by affecting JA signaling [50]. CaDILZ1, a member of the *Capsicum annuum* bZIP protein family, exhibited drought-tolerant phenotypes via ABA-mediated drought stress signaling in *Arabidopsis* plants [51]. In our study, the expression of *GmbZIP152* was increased by salt, drought, and heavy metal (Figure 1B–E), suggesting that *GmbZIP152* is involved in the abiotic response. Our phenotypic analysis showed that the tolerance of salt, drought, and heavy metal were significantly increased in *OE-GmbZIP152* plants compared to WT (Figure 3 and Figure S3). From the results of qRT-PCR, the expression of ABA-responsive marker genes, *AtABI2*, *AtABI5*, and *AtABF1*, were increased under salt and drought (Figure 5). These results suggested that *GmbZIP152* responds to biotic and abiotic stresses by involving the hormone-responsive pathways.

Reactive oxygen (ROS) is the key signaling molecule produced under biotic and abiotic stress conditions and triggers various plant defense responses [52]. Studies have shown that plants will produce excessive ROS (H_2_O_2_ and O^2−^) after being subjected to a different stress condition, which affects the growth, development, and yield of plants [53,54]. To neutralize excess ROS under stress conditions, plants have synthesized several antioxidants, such as SOD, POD, and CAT, to scavenge ROS and restore cellular redox homeostasis [55,56,57,58,59]. Our results showed that the H_2_O_2_ content of *OE-GmbZIP152* plants was less than WT plants under *S. sclerotiorum* treatment. And the activities of CAT, SOD, and POD were activated in *OE-GmbZIP152* plants under salt, drought, copper, and cadmium stress (Figure 4A–C), indicating that enhanced ROS scavenging capability of *OE-GmbZIP152* plants in comparison to WT plants. To understand the regulatory function of *GmbZIP152*, we check the transcript levels of antioxidant genes (*AtCAT1*, *AtSOD*, and *AtPOD1*). The qRT-PCR results showed that the expression levels of these genes were higher in the *OE-GmbZIP152* leaves compared to WT leaves under stress conditions. Therefore, we hypothesized that *GmbZIP152* regulates the activity of the ROS-scavenging enzyme by affecting the transcript level of the antioxidant genes.

Moreover, we performed qRT-PCR to investigate the expression level of several stress-related genes in *OE-GmbZIP152* plants and WT controls (Figure 5). In our study, the expression levels of various stress-responsive genes, for example, *AtHARDY*, *AtDREB2A*, *AtPUB19*, *AtCOPT1*, *GmSYT2*, *AtGSH1*, and *AtPCS1* et al., were significantly higher in *OE-GmbZIP152* plants than those in WT plants under normal or stress conditions. These findings indicated that *GmbZIP152* affects plant stress tolerance by altering the expression of stress-related genes.

To further explore the stress resistance of *OE-GmbZIP152* in soybean, we screened out hormone-associated stress-related through previous reports. For example, Ding et al. stated that *NPR3*, a SA receptor plays a key role in transcriptional regulation of SA-induced defense gene expression [60]. *PR2* comprised an important component in the SA defense signaling pathway as an SA-responsive gene [61]. COI1, encoding a F-box protein, was involved in regulating the wounding response through JA-related processes [62]. *GmRD22* is up-regulated by drought-, salinity-stress and exogenously supplied ABA [63]. GmBIP, is a molecular chaperone that increases drought tolerance in soybean by delaying leaf senescence [64]. *GmSOD* participates in encoding the antioxidant enzyme [65]. In the result of ChIP-qPCR, GmbZIP152 directly binds to the promoter of GmABI5 and GmSOD. These genes were also differentially regulated in the *OE-GmbZIP152* lines (Figure 5). The ChIP-qPCR analysis showed that GmbZIP152 directly binds to the promoter of hormone-, stress-, and antioxidant enzyme-related genes in soybean (Figure 6). These results are similar to the function of *GmbZIP152* in *Arabidopsis*, indicating that *GmbZIP152* may as a positive regulator in soybean response to abiotic and biotic stresses.

**Figure 7:**
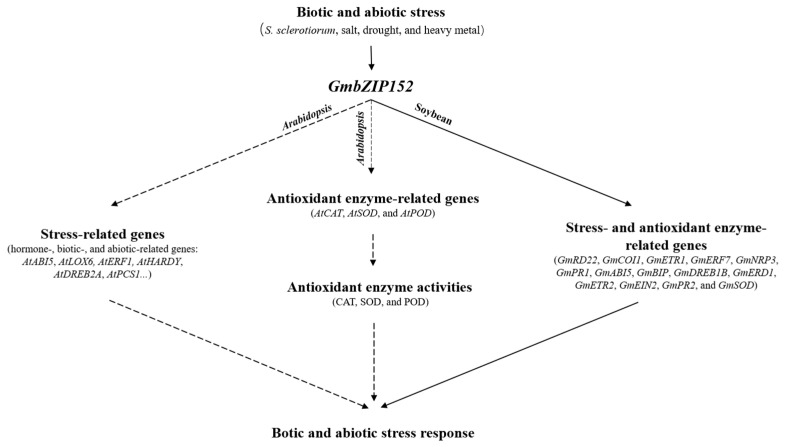
A schematic model of *GmbZIP152* mediated biotic- and abiotic-stress tolerance in transgenic *Arabidopsis*. *GmbZIP152* positively modulates the biotic- and abiotic-stress tolerance: *GmbZIP152* positively regulates the expression of antioxidant enzyme, hormone, biotic, and abiotic-related genes. The dashed lines indicate indirect regulation, and solid lines indicate direct regulation. The arrows indicate induction or positive modulation.

In general, our results show that *GmbZIP152* is a multi-functional transcription factor, which involves in disease defense and abiotic stress tolerance by regulating phytohormone-responsive genes, biotic and abiotic stress-responsive genes, and the antioxidant enzyme activities (Figure 7). As a result of the ChIP-qPCR, GmbZIP152 directly binds to the promoter of many hormone-related genes (Figure 6). It suggested that *GmbZIP152* can directly regulate the tolerance of biotic and abiotic by the hormone signaling pathway. And *GmbZIP152* directly regulates the antioxidant enzyme activities of SOD by *GmSOD* (Figure 6), but the antioxidant enzyme activities of CAT and POD are indirectly affected. In addition, *GmbZIP152* improve the biotic and abiotic stress tolerance by indirectly regulating the biotic- and abiotic-related genes (Figure 5). However, much more work needs to be conducted to deeply understand the other components and molecular mechanisms that interact with the functions of the underlying *GmbZIP152* under biotic and abiotic stress in the future.

## 4. Materials and Methods

### 4.1. GmbZIP152 Gene Isolation, Vector Construction, and Arabidopsis Transformation

We used an RNA extraction kit (Omega Bio-Tek, Shanghai, China) to extract total RNA from the leaves of William 82 (*Glycine max*). The cDNA was synthesized using PrimerScript^TM^RTase (TaKaRa Biotechnology, Beijing, China), according to the manufacturer’s instructions. The full length of grape *GmbZIP152* (*Glyma.19G216200*) open reading frame (ORF) was amplified by PCR using gene-specific primers (Supplemental Table S1). The PCR product was cloned into the pGWB 605 vector, and the plasmid (*pGWB605-GmbZIP152*) was sequenced to confirm sequence fidelity.

The plasmid with the targeted gene was introduced into *A.tumefaciens* strain GV3101 via electroporation and transformed into *A. thaliana* by using the floral dip method [66]. T_0_ seeds were harvested and sown on the soil. After one week, we used 0.1% glufosinate ammonium (LIER-Chemical, Mianyang, China) to screen transgenic lines. We selected two lines (*GmbZIP152-2* and *GmbZIP152-5*) from 10 independent lines, and three-week-old T_3_ homozygous lines were generated and used for all further experiments. The relative expression level of *GmbZIP152* was examined in *Arbidopsis* using qRT-PCR (Figure S5).

### 4.2. Cis-Element Analysis of GmbZIP152 Promoters

The 2.5 kb upstream sequence of the *GmbZIP152* was retrieved from the Phytozome V12.1 (https://phytozome.jgi.doe.gov/pz/portal.html, accessed on 30 January 2020) and then submitted to Plant Cis-Acting Regulatory Element (PlantCARE, http://bioinformatics.psb.ugent.be/webtools/plantcare/html/, accessed on 30 January 2020) [67] to detect the presence of the following five regulatory elements [68] (Supplemental Table S2): G-box (CACGAC); TC-rich repeats (G/ATTCTCT); MYB (CAACTG); TCA-element (CCATCTTTTT); CGTCA-motif (CGTCA).

### 4.3. Plant Growth Conditions

*Arabidopsis thaliana* plants were grown on soil mixture 2:1 (*v*/*v*) peat moss: perlite in plastic pots in a greenhouse under the following conditions: 22 °C, 65% humidity, and a 16-h light/8-h dark photoperiod. Soybean (William 82) seeds were sown in soil and the photoperiod was 16 h light/8 h dark at 25 °C in a greenhouse.

Seeds from each of two selected T_3_ *OE-GmbZIP152* lines and WT were sterilized in 95% ethanol for 5 min and then treated with 75% ethanol for 15 min, followed by four washes with sterilized distilled water. The seeds were then plated on 1/2 MS medium. After 7-days, transgenic and WT seedlings were transferred into the compost soil and used for further experiments.

### 4.4. Stress Tolerance Assays and Measurements of Physiological Indices

In preparation for the germination assays, ~100 seeds were surface-sterilized and sown on MS medium supplemented with different concentrations of NaCl (100 mM and 150 mM), mannitol (250 mM and 300 mM), CuSO_4_ (50 uM and 100 uM), CdSO_4_ (50 uM and 100 uM), ETH (400 uM), JA (200 uM), and ABA (1.0 uM). Seeds were vernalized at 4 °C for 3 days before growing in a growth chamber. The root length and fresh weight were measured on day 7 after growing.

For the plant growth assays, 7-day-old *OE-GmbZIP152* and WT seedlings were transferred into the compost soil. We treated three-week-old WT and *OE-GmbZIP152* plants with NaCl (100 mM and 150 mM), mannitol (250 mM and 300 mM), CuSO_4_ (50 uM and 100 uM), and CdSO_4_ (50 uM and 100 uM) for 18 days and measured the plant height. All experiments were repeated three times.

To explore the expression profile of *GmbZIP152*, two-week-old soybean seedling leaves were infected with *S. sclerotiorum* and seedlings were treated with 150 mM NaCl for salt conditions, 400 mM mannitol for drought conditions, 150 uM CuSO_4_, and 150 uM CdSO_4_ for heavy metal conditions, 400 uM ETH, 150 uM JA, 1.0 uM ABA, and 250 uM SA. In addition, the eight-week-old mature soybean was treated with NaCl, mannitol, CuSO_4_, CdSO_4_, ETH, JA, ABA, and SA. The leaves detected the expression level of *GmbZIP152*.

The three-week-old plants were treated with SOD, CAT, and POD Activity Detection kit (Solarbio, Beijing, China) for 24 h to measure the physiological indices, according to the manufacturer’s instructions.

### 4.5. Pathogens and Inoculation Procedures

For *S. sclerotiorum* treatments, the fungal strains preserved at 4 °C were subcultured on potato dextrose agar medium for two days first. Then, we excised the new marginal hyphae using a 7 mm puncher and closely upended them onto the surface of leaves from three-week-old plants. The inoculated leaves were placed in a square petri dish and transferred into a growth chamber that allowed disease symptoms development. The disease spot area was measured after 2 days using ImageJ [69]. All experiments were repeated three times.

### 4.6. RNA Extraction and Quantitative Real-Time PCR

Samples were collected after treatment, and two independent seedlings were randomly harvested and frozen by liquid nitrogen immediately, then stored at −80 °C store for RNA extraction. Total RNA was extracted using the RNA plant extraction Kit (Omega Bio-Tek, Shanghai, China) following the manufacturer’s protocol. The obtained RNA concentrations range from 100 to 500 ng/μL, and the OD260/OD280 ratios ranged from 1.8 to 2.0. According to the supplier’s instructions to use AMV reverse transcriptase (Takara), 1 μg of purified total RNA was reverse transcribed to cDNA in a 20 μL reaction volume [70]. Subsequent quantitative real-time PCR was performed with gene specific primers according to the manufacturer’s instructions on the Bio-Rad Real-time PCR system (Foster City, CA, USA). The specific primers used in this experiment are given in Supplemental Table S3. The PCR program was set: 95 °C for 30 s; 40 cycles of 95 °C for 5 s and 60 °C for 34 s; 95 °C for 15 s. In each case, three technical replicates and at least three independent biological replicates were performed [20,71]. Relative expression was calculated using the 2^-ΔΔCt^ method [72]. Data were analyzed using a one-way analysis of variance (ANOVA) (Supplemental Tables S4 and S5).

### 4.7. Transient GmbZIP152 Expression Assay

GV3101 carrying the *pGWB 605-GmbZIP152* vector was cultured to OD_600_ = 1.0 induction medium [10 mM ethanesulfonic acid (pH 5.7), 10 mM MgCl_2_, 200 mM acetosyringone] and diluted to OD_600_ = 0.8. This was injected into two-week-old soybean leaves (*William 82*) and transferred to soybean plants into a growth chamber for 2 days. The injected leaves were then harvested for further use.

### 4.8. Chromatin Immunoprecipitation (ChIP) Analysis

For the Chromatin immunoprecipitation (ChIP) experiment, approximately 4 g of two-week-old soybean leaves transiently overexpressing *GmbZIP152* were used. Samples were formaldehyde cross-linked [69]. Crosslinked chromatin was fragmented with 0.2 units of micrococcal nuclease (Sigma, St. Louis, MO, USA) in 1 mL of MNase digestion buffer [10 mM Tris-HCl (pH 8.0), 50 mM NaCl, 1 mM β-mercaptoethanol, 0.1% NP-40, 1 mM CaCl_2_, and protease inhibitor cocktail (Roche)]. Chromation stopped digestion using 5 mM EDTA. ChIP was performed using an anti-GFP antibody (Abcam, Cambridge, U.K.). Relative enrichment of associated DNA fragments was analyzed by qPCR. All primers used in the ChIP experiments are given in Supplemental Table S6. Each ChIP experiment was repeated twice, and the presented data are from one representative experiment.

## 5. Conclusions

In this study, we cloned and characterized soybean *GmbZIP152*. Our results showed that overexpression of *GmbZIP152* will increase resistance to disease infection and tolerance of abiotic stresses by regulating phytohormone-responsive genes, biotic and abiotic stress-responsive genes, and the antioxidant enzyme activities. These findings deepen the understanding of the role of the soybean *GmbZIP152* transcription factor in the molecular mechanisms of complex biotic and abiotic stress. They provided a theoretical basis for the functional characterization of *GmbZIP152* genes in different plant species.

## Data Availability

All data analyzed during this study are included in this article and its additional files.

## Supplementary Materials

The supporting information can be downloaded at: https://www.mdpi.com/article/10.3390/ijms231810935/s1.
