# Supplementary material for: GmbZIP152, a Soybean bZIP Transcription Factor, Confers Multiple Biotic and Abiotic Stress Responses in Plant
Source: Int J Mol Sci. 2022 Sep 19;23(18):10935. doi: 10.3390/ijms231810935 (PMC9505269; doi:10.3390/ijms231810935)
Supplement: Supplementary file 1 [file ijms-23-10935-s001.zip › ijms-1886684-supplementary/Supplemental Figures.pdf]

GmbZIP152, a Soybean bZIP Transcription Factor, Confers Multiple Biotic and Abiotic Stress Responses in Plant

Mengnan Chai [1,†], Rongbin Fan [1,†], Youmei Huang [2], Xiaohu Jiang [1], Myat Hnin Wai [1], Qi Yang [1], Han Su [2], Kaichuang Liu [2], Suzhuo Ma [2], Zhitao Chen [1], Fengjiao Wang [1], Yuan Qin [1,2,3,4,*] and Hanyang Cai [2,*]

Supplemental Figures

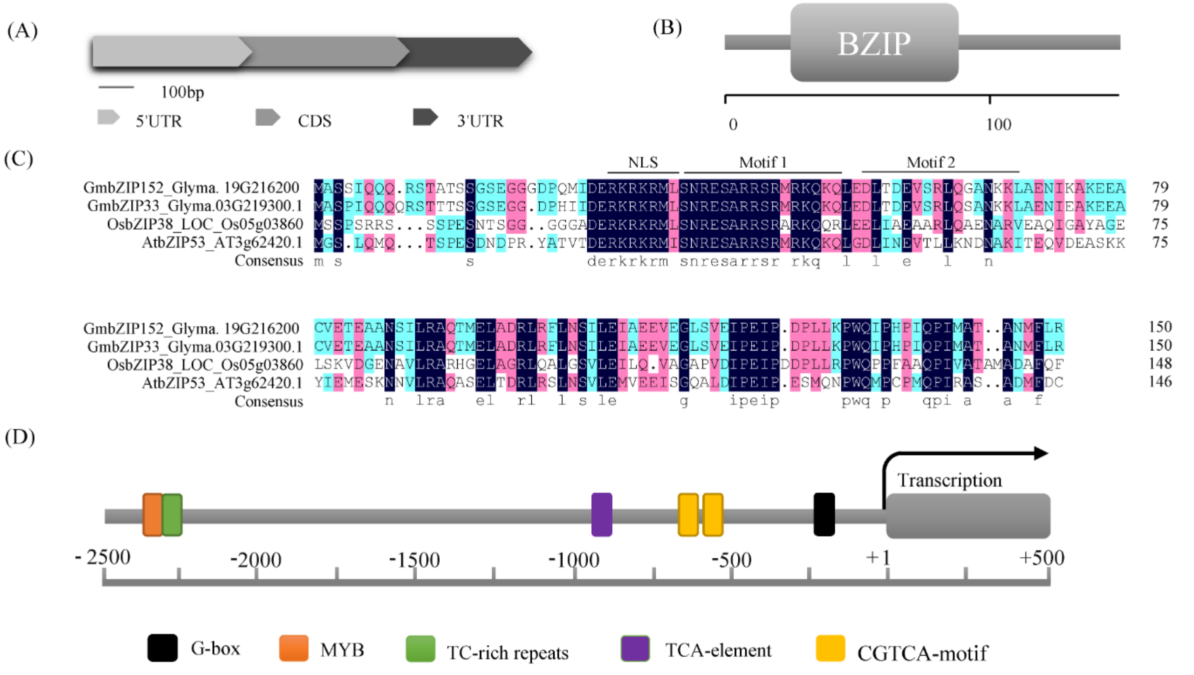

Supplemental Figure S1. Bioinformatics analysis of *GmbZIP152*. (A) The gene structure analysis. (B) The conserved bZIP domain analysis. (C) Multiple alignments of the conserved DNA-binding region and leucine zipper region between GmbZIP33, OsbZIP38, and AtbZIP53. (D) The stress-related *cis*-elements in *GmbZIP152* promoter.

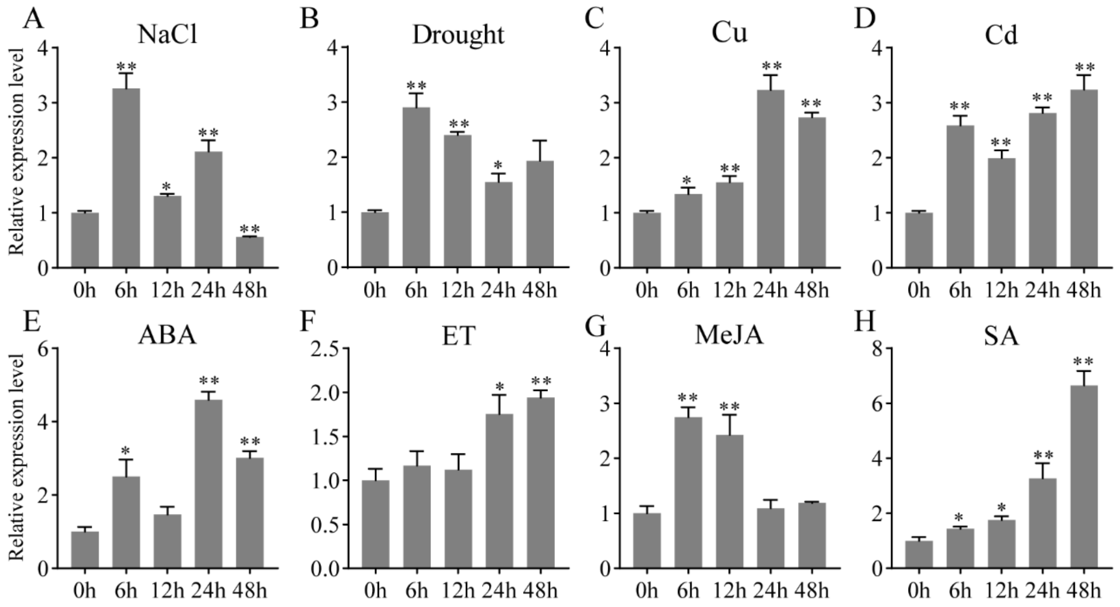

Supplemental Figure S2. Expression pattern of the *GmbZIP152* gene revealed by quantitative PCR analysis from different treatments in mature soybean. (A)-(D) *GmbZIP152* expression in response to various abiotic stress treatments (150 mM NaCl, 400 mM Mannitol, 150 μM CuSO4, and 150 μM CdSO4). (E)-(H) *GmbZIP152* expression in response to various hormone treatments [1.0 μM Abscisic acid (ABA), 150μM Methyl jasmonic acid (MeJA), 400 μM Ethylene (ETH), and 250 μM Salicylic acid (SA)]. Errors bars indicate ± SD of three biological replicates. Asterisks indicate significant differences for the indicated comparisons based on a Students' t-test (**$p < 0.01$; $0.01 <$ *$p < 0.05$).

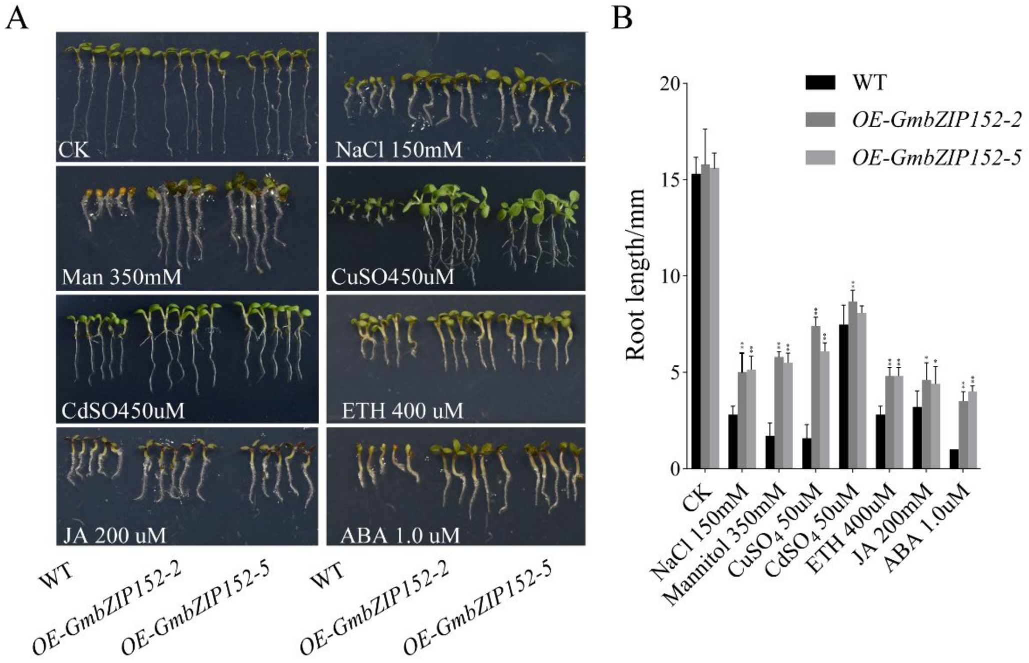

Supplemental Figure S3. Phenotypic analysis of *GmbZIP152* transgenic *Arabidopsis* plants in response to salt, drought, heavy metal, plant hormones treatment in *Arabidopsis*. (A) The diagram of root length comparison (scale bar, 1 cm). (B) Calculation of the seedlings' root length. Control check (CK), Abscisic acid (ABA), Methyl jasmonic acid (MeJA), Ethylene (ETH), and Salicylic acid (SA). *GmbZIP152* transgenic *Arabidopsis* plants (*OE-GmbZIP152-2* and *OE-GmbZIP152-5*, two independent transgenic lines). Errors bars indicate ± SD of three biological replicates. Experiments were repeated three times. Asterisks indicate significant differences for the indicated comparisons based on a Students' t-test (**p< 0.01; 0.01<*p<0.05).

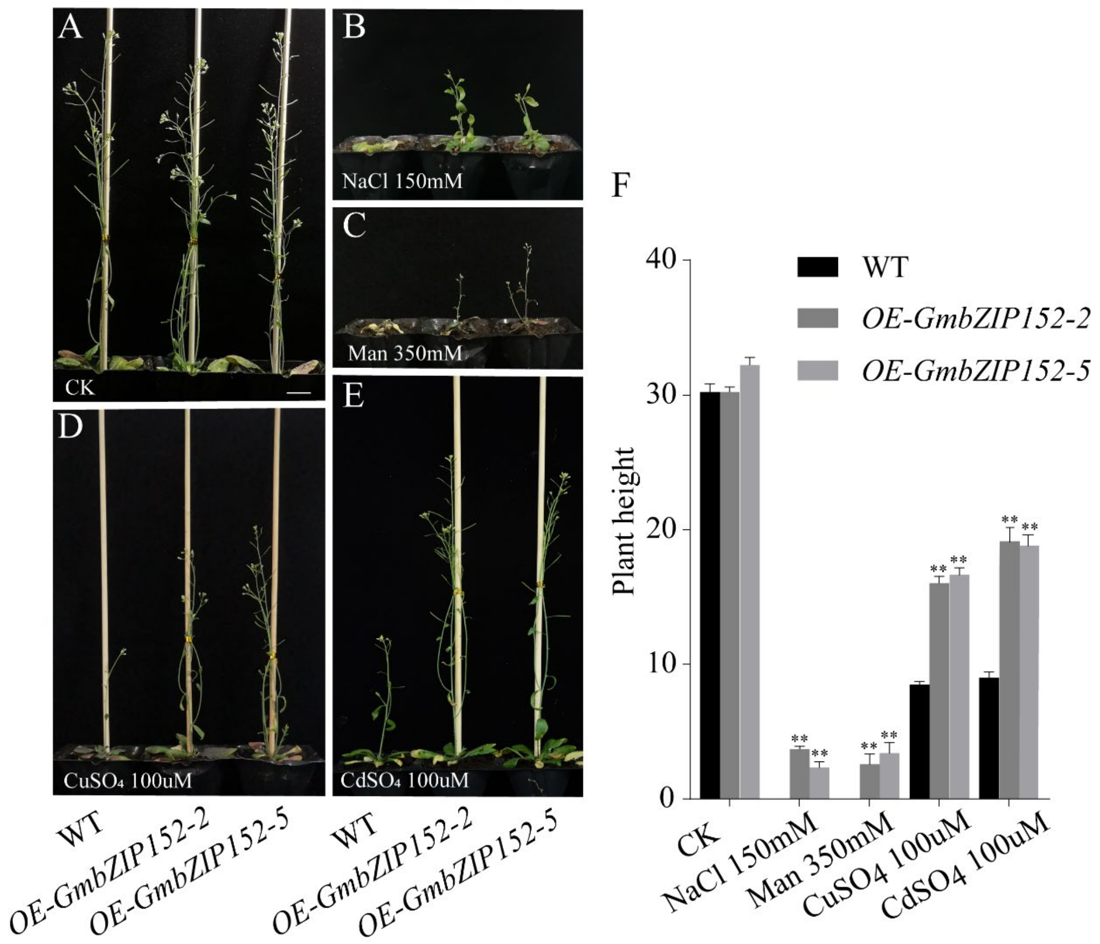

Supplemental Figure S4. Phenotypic analysis of *GmbZIP152* transgenic *Arabidopsis* plants in response to salt, drought, heavy metal, plant hormones treatment in *Arabidopsis*. (A)-(E) The diagram of root length comparison. (F) Calculation of the plant height. Control check (CK), *GmbZIP152* transgenic *Arabidopsis* plants (*OE-GmbZIP152-2* and *OE-GmbZIP152-5*, two independent transgenic lines). Errors bars indicate ± SD of three biological replicates. Asterisks indicate significant differences for the indicated comparisons based on a Students' t-test (**p$<$0.01; 0.01$<$*p$<$0.05).

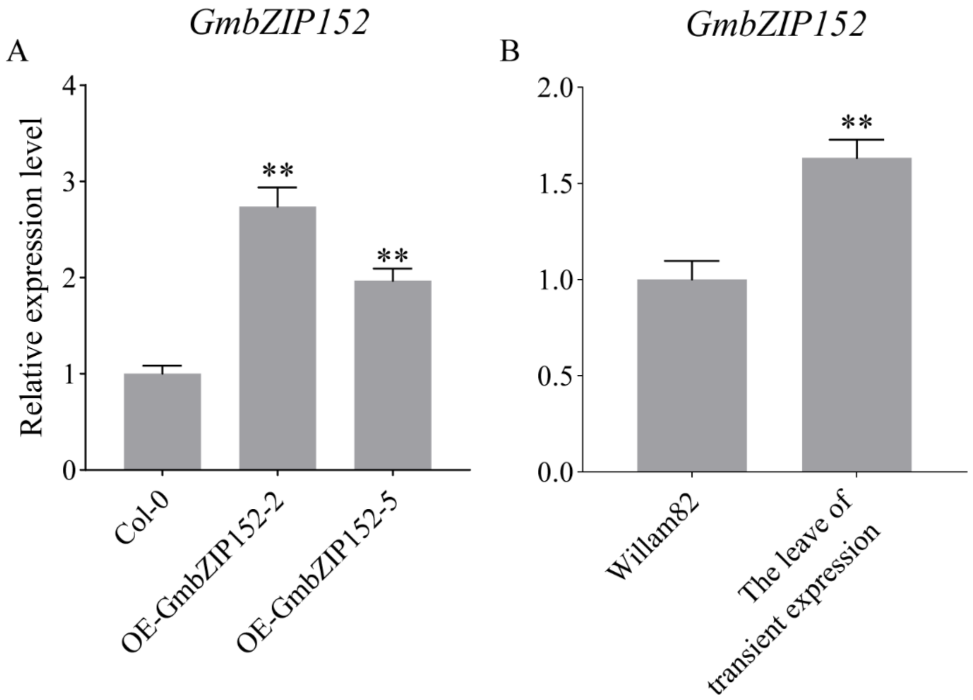

Supplemental Figure S5. Analysis of expression level of *GmbZIP152* in transgenic *Arabidopsis* plants and in the transient expression assay. (A) Relative expression level of *GmbZIP152* in Col-0, *OE-GmbZIP152-2*, and *OE-GmbZIP152-2*. (B) Relative expression level of *GmbZIP152* in William82 and the leave of transient expression. *GmbZIP152* transgenic *Arabidopsis* plants (*OE-GmbZIP152-2* and *OE-GmbZIP152-5*, two independent transgenic lines). The error bars indicate ±SD (n=3 replicates). Asterisks indicate significant differences for the in-dicated comparisons based on a Students' t-test (**p＜0.01; 0.01 ＜*p＜0.05).
